# Sequential Probability Ratio Testing with Power Projective Base Method Improves Decision-Making for BCI

**DOI:** 10.1155/2017/2948742

**Published:** 2017-11-14

**Authors:** Rong Liu, Yongxuan Wang, Xinyu Wu, Jun Cheng

**Affiliations:** ^1^Biomedical Engineering Department, Dalian University of Technology, Dalian, Liaoning 116024, China; ^2^Affiliated Zhongshan Hospital of Dalian University, Dalian, Liaoning 116001, China; ^3^Shenzhen Institutes of Advanced Technology, Chinese Academy of Sciences, Shenzhen 518055, China

## Abstract

Obtaining a fast and reliable decision is an important issue in brain-computer interfaces (BCI), particularly in practical real-time applications such as wheelchair or neuroprosthetic control. In this study, the EEG signals were firstly analyzed with a power projective base method. Then we were applied a decision-making model, the sequential probability ratio testing (SPRT), for single-trial classification of motor imagery movement events. The unique strength of this proposed classification method lies in its accumulative process, which increases the discriminative power as more and more evidence is observed over time. The properties of the method were illustrated on thirteen subjects' recordings from three datasets. Results showed that our proposed power projective method outperformed two benchmark methods for every subject. Moreover, with sequential classifier, the accuracies across subjects were significantly higher than that with nonsequential ones. The average maximum accuracy of the SPRT method was 84.1%, as compared with 82.3% accuracy for the sequential Bayesian (SB) method. The proposed SPRT method provides an explicit relationship between stopping time, thresholds, and error, which is important for balancing the time-accuracy trade-off. These results suggest SPRT would be useful in speeding up decision-making while trading off errors in BCI.

## 1. Introduction

Noninvasive brain-computer interface (BCI) based on the electroencephalogram (EEG) offers a new means of communication to locked-in or paralyzed patients [1, 2] and controlling a prosthesis [3, 4] without reliance on the usual neuromuscular pathways. The critical challenge of BCI technology is to classify the brain signals and mental tasks accurately and fast. However, the EEG recorded from the scalp has the characteristics of low strength, low SNR (signal noise ratio), and the EEG difference under different mental tasks is not significant. Therefore, various pattern recognition algorithms were used in BCI system to extract and classify EEG features.

Event-related desynchronization/synchronization (ERD/ERS) patterns of motor imagery are effective features for EEG-based BCI systems. The experiments show that the phenomenon of ERD/ERS varies among individuals. Therefore, a pattern recognition algorithm should be used to facilitate decoding “motor intent,” both to find subject-specific EEG features that maximize the separation between the patterns generated by executing the mental tasks and to train classifiers that minimize the classification error rates of these specific patterns. Currently, feature extraction for discrimination of left- and right-hand motor imagery EEG is usually based on EEG band power (BP). For example, autoregression (AR) model [5], discrete Fourier transformation (DFT) [6], and wavelet transforms (WT) [7] have been used to extract EEG features for classification. The wavelet method is one of the most effective algorithms. However, the success of wavelet application greatly depends on the proper selection of subject-specific parameters. Actually, the wavelet transform can be considered as projecting the EEG onto a wavelet basis and the band power as the modulus values of projective coefficients. Inspired by the wavelet method, we introduce a new feature extraction method based on power projective bases to classify EEGs without constrain of wavelet forms.

Moreover, the ability to make rapid decisions based on transient stimuli is a unique aspect of our brains' capacity to process information. Broadly speaking, signal detection theory (SDT) and sequential analysis (SA) are two branches of mathematical models that provide a theoretical framework for understanding how decisions are made [8]. SDT converts a single observation into a categorical choice. According to different decision rules, there are different testing approaches to this problem [9]. For example, Bayesian decision theory is derived by minimizing the posterior expected loss, while Neyman-Pearson (NP) criterion seeks to find the best error probability (*α*) level test. Like most statistical classification methods, for example, linear discriminant analysis (LDA) and support vector machines (SVM), the classification error is the only characteristic of the SDT decision strategies. The necessary number of observation samples determined by the criteria could be very large, which is especially impractical for BCI applications. To control brain-actuated devices, such as robotics and neuroprostheses, both fast decision-making and a stable control signal with a minimal error rate are important [10, 11]. Therefore, recent attentions have been paid to the variable-length sequential sampling model.

A systematic theory of optimal stopping emerged with the work by Wald on the optimality of the sequential probability ratio test (SPRT) [12]. The SPRT achieves a desired error rate with the smallest number of samples, on average. Therefore, in this paper, we introduce a new feature extraction method based on power projective base to classify the EEGs by combining the sequential probability ratio test (SPRT) approach to obtain a continuous dynamic estimate of brain state with accuracy and decision speed balance.

## 2. Methodology

### 2.1. Data Description

The EEG data used in this work were obtained from thirteen subjects from BCI Competitions II, III, and IV. The task was performed based on left- and right-hand motor imagination.

#### 2.1.1. Dataset III from BCI Competition II

This dataset contains EEG data from one subject (S1) [13]. The data were recorded from three channels (C3, Cz, and C4) and sampled at 128 Hz. The data consist of 140 labelled and 140 unlabelled trials with an equal number of left- and right-hand trials. Each trial has a duration of 9 s, where a visual cue (arrow) is presented pointing to the left or the right after 3 s preparation period followed by a 6 s motor imagery (MI) task.

#### 2.1.2. Dataset IIIb from BCI Competition III

The second dataset contains EEG data recorded over the channels C3 and C4 from three subjects (S2, S3, S4) with some corrections [14]. The data were sampled at 125 Hz. Training and testing sets were available for each subject. Except for the subject O3 that has only just 320 trials for each set, the subjects S4 and X11 contain 540 labelled and 540 unlabelled trials. Each trial has duration of 7 s which consists of 3 s for preparation period and 1 s for a visual cue presentation, followed by another 3 s for the imagination task.

#### 2.1.3. Dataset IIb from BCI Competition IV

This dataset contains EEG data from nine subjects (S5–S13) [15]. The data were recorded from three bipolar channels (C3, Cz, and C4) and 3 EOG channels. The sample frequency was 250 Hz. Training and testing set was available for each subject. Each subject participated in two screening sessions without feedback and three online feedback sessions with smiley feedback. The trials without feedback had duration of 7 s, and a visual cue was presented for 1.25 s followed by another 4 s for the imagination task. The trials with feedback had duration of 7.5 s, and a visual cue was presented for 4.5 s until the end of motor imagination.

### 2.2. Feature Extraction Method Based on Power Projective Base

Motor imagery can be regarded as mental rehearsal of a motor act without any obvious motor output. Recent studies show that when performing motor imagination, *μ* (8–13 Hz) and *β* (18–30 Hz) rhythms are found to reveal event-related desynchronization and synchronization (ERD/ERS) over sensorimotor cortex just as when one performs motor tasks. Due to nonstationary effects having often been observed in brain signals, we proposed a power projective base method to extract classification features from C3 and C4 channels. This method improves the classification accuracy by maximizing the difference of the average projective power between two-class signals. Specifically, the solution of the projective bases can be achieved by generalized eigenvalue decomposition for each subject.

Let **X**_*k*_ ∈ *R*^*M*×*N*_*k*_^ be the training dataset from one channel, where *k* ∈ {*L*, *R*} denotes the left- or right-hand motor imagery tasks, *M* denotes the sampling points, and *N*_*k*_ is the number of trials. Moreover, let **u** ∈ *R*^*M*^ be the projective basis and ‖**u**‖ = 1. The projective power of signal **x**_*kj*_, *j* = 1,2,…, *N*_*k*_ on the projective basis **u** is

(1) $$\begin{array}{c} z_{kj}\left(\;u\;\right)=\left(\;u^{T}x_{kj}\;\right)\cdot \left(\;x_{kj}^{T}u\;\right). \end{array}$$

So the mean projective power ${\overset{-}{z}}_{k}(u)$ can be calculated as

(2) z−ku=1Nk∑j=1NkuTxkj·xkjTu=uT·XkXkTNk·u=uTRku,

where **R**_*k*_ = **X**_*k*_**X**_*k*_^*T*^/*N*_*k*_ is the autocorrelation matrix and it is usually positively definite.

To formulate the objective function to be the ratio of the two-class average projective powers,

(3) $$\begin{array}{c} F\left(\;u\;\right)=\frac{{\overset{-}{z}}_{L}}{{\overset{-}{z}}_{R}}=\frac{u^{T}R_{L}u}{u^{T}R_{R}u}. \end{array}$$

By maximizing or minimizing *F*(**u**) to be *F*_max_ or *F*_min_, the corresponding eigenvector **u**_max_ or **u**_min_ is the optimal projective base to be solved. The optimization of (3) could be solved by taking a generalized eigenvalue decomposition method. First of all, we can get the following decomposition as

(4) TTRLT=Λ=diag⁡λ1,…,λM,λi≥λj>0  ∀i<j,TTRRT=I,

where **T** = [**t**_1_,…, **t**_*M*_] is the generalized eigenvector matrix and *λ*_*i*_ is the generalized eigenvalue. Therefore, the ratio of mean projection power *F*(**u**) turns to

(5) Fu=uTT−TΛT−1uuTT−TT−1u=uTT−T·Λ·T−1uuTT−T·T−1u≜vTΛvvTv=λ1v12+⋯+λMvM2v12+⋯+vM2,

where **v** = **T**^−1^**u**. Since

(6) λ1v12+⋯+λMvM2v12+⋯+vM2≤λ1v12+⋯+λ1vM2v12+⋯+vM2=λ1,λ1v12+⋯+λMvM2v12+⋯+vM2≥λMv12+⋯+λMvM2v12+⋯+vM2=λM

*F*(**u**) has the maximum value *F*_max_ = *λ*_1_ and minimum value *F*_min_ = *λ*_*M*_. Obviously, the corresponding vectors **v** can be obtained by

(7) vmax=1,0,…,0T,vmin=0,…,0,1T.

Then, we have

(8) umax=T·1,0,…,0T,umin=T·0,…,0,1T

which means that **u**_max_ and **u**_min_ are the first column **u**_1_ and last column **u**_*M*_ of **T**, respectively. Choosing **u**_max_ or **u**_min_ as the projective base depends on which is larger between *λ*_1_ and 1/*λ*_*M*_. The projective power for the signals of the channels C3 and C4 onto their own projective bases is then stacked together into a 2-dimensional feature vector **z** = [*e*_C3_, *e*_C4_]^T^.

### 2.3. SPRT Classification Method

Sequential analysis is a statistical decision model that assumes decisions are formed by continuously sampling information until the response criterion is satisfied. Once a boundary has been reached, the decision process is concluded and a response is elicited. The number of observations needed for a decision is not determined in advance of the experiment, but by the observations obtained during the test. The data should be fed to the SPRT algorithm sequentially, so we divide each trial into segments with overlap and each one has the same length as that of the projective base used in feature extraction.

Taking into account the nonstationarity of the EEG sampling information, we assume that the probability distribution of the *i*th segment feature **z**_*i*_ for class *k* is *g*_*k*_(**z**_*i*_), *k* ∈ {*L*, *R*}, and then the probability ratio for the *i*th segment is

(9) $$\begin{array}{c} l_{i}\left(\;z_{i}\;\right)=\frac{g_{Li}\left(z_{i}\right)}{g_{Ri}\left(z_{i}\right)} \end{array}$$

and the evidence accumulation turns to

(10) $$\begin{array}{c} l_{q}\left(\;z_{1},z_{2},\ldots ,z_{q}\;\right)=\frac{\prod_{i=1}^{q}g_{Li}\left(z_{i}\right)}{\prod_{i=1}^{q}g_{Ri}\left(z_{i}\right)}, \end{array}$$

where *q* is the number of accumulated segments. Assuming the segments are independent for computation convenience [12], we have

(11) $$\begin{array}{c} l_{q}\left(\;z_{1},z_{2},\ldots ,z_{q}\;\right)=\frac{g_{L}\left(z_{1},z_{2},\ldots ,z_{q}\right)}{g_{R}\left(z_{1},z_{2},\ldots ,z_{q}\right)}, \end{array}$$

where *g*_*k*_(**z**_1_, **z**_2_,…, **z**_*q*_), *k* ∈ {*L*, *R*} is the join probability distribution of *q* dimensional vector (**z**_1_, **z**_2_,…, **z**_*q*_). This assumption is violated by our data in practice.

The decision rule with two thresholds *η*_*L*_ and *η*_*R*_ is

(12)  if  lqz1,z2,…,zq≥ηL,k^=L if  lqz1,z2,…,zq≤ηR,k^=R if  ηR<lqz1,z2,…,zq<ηL,compute  lq+1.

With two thresholds, we have the option to increase *η*_*L*_ or decrease *η*_*R*_ which will increase the probability to make a correct decision (by waiting to accumulate more data or evidence) but decrease the probability of making a wrong decision (by delaying the decision). The error probabilities are defined as

(13) pL ∣ R=Pk^=L ∣ k=R,pR ∣ L=Pk^=R ∣ k=L.

If *l*_*q*_(**z**_1_, **z**_2_,…, **z**_*q*_) ≥ *η*_*L*_ is satisfied, we define the corresponding space of vector (**z**_1_, **z**_2_,…, **z**_*q*_) to be *Z*_*L*_. With (11), we have

(14) $$\begin{array}{c} g_{L}\left(\;z_{1},z_{2},\ldots ,z_{q}\;\right)\geq \eta_{L}\cdot g_{R}\left(\;z_{1},z_{2},\ldots ,z_{q}\;\right). \end{array}$$

Equation (14) is then integrated in *Z*_*L*_ yielding

(15) $$\begin{array}{c} \overset{}{\underset{Z_{L}}{\int }}g_{L}\left(\;z_{1},z_{2},\ldots ,z_{q}\;\right)\geq \eta_{L}\cdot \overset{}{\underset{Z_{L}}{\int }}g_{R}\left(\;z_{1},z_{2},\ldots ,z_{q}\;\right). \end{array}$$

That is,

(16) $$\begin{array}{c} 1-p_{R\;|\;L}\geq \eta_{L}\cdot p_{L\;|\;R}. \end{array}$$

Analogous reasoning for *l*_*q*_(**z**_1_, **z**_2_,…, **z**_*q*_) ≤ *η*_*R*_ yields

(17) $$\begin{array}{c} p_{R\;|\;L}\leq \eta_{R}\cdot \left(\;1-p_{L\;|\;R}\;\right). \end{array}$$

Thus, the two detection thresholds *η*_*L*_ and *η*_*R*_ are related to the error probabilities by

(18) pL ∣ R≤1−pR ∣ LηL≤1ηL,pR ∣ L≤1−pL ∣ R·ηR≤ηR.

The two kinds of error probabilities can be lowered by either increasing *η*_*L*_ or decreasing *η*_*R*_. However, due to the limited number of segments, the indecision ratio will increase as the error probability *p*_*L*∣*R*_ or *p*_*R*∣*L*_ is decreased. Therefore, we may not obtain the optimal result by simply increasing *η*_*L*_ or decreasing *η*_*R*_. The suitable *η*_*L*_ and *η*_*R*_ could be achieved by the following optimization criteria.

Under the assumption that the features follow a Gaussian distribution, we can take logarithm on both sides of (10) to obtain the log probability ratio (log PR), which leads a sequential probability ratio test (SPRT) as

(19) Lq=∑i=1q12log⁡SRiSLi+DM2zi,mRi−DM2zi,mLi≜∑i=1qJi,

where $D_{M}\left(z_{i},m_{ki}\right)=\sqrt{\left(z_{i}-m_{ki}{)}^{T}S_{ki}^{-1}(z_{i}-m_{ki}\right)}$ is the Mahalanobis distance and *J*_*i*_ is the log PR of the *i*th segment.

We can derive the average *J*_*i*_ for each class:

(20) EJi ∣ k=L=12log⁡SRiSLi+tr⁡SLi+mLi−mRi·mLi−mRiT·SRi−1−I,EJi ∣ k=R=12log⁡SRiSLi−tr⁡SRi+mRi−mLi·mRi−mLiT·SLi−1−I.

For any given threshold pair *η*_*L*_ and *η*_*R*_, the number of accumulated segments to make a correct decision, that is, stopping time, for class *k*, is *E*(*q*∣*k*) which satisfies

(21) Eq ∣ k=L=infn⁡∑i=1nEJi ∣ k=L≥1−ηR·log⁡ηL+ηR·log⁡ηREq ∣ k=R=infn⁡∑i=1nEJi ∣ k=R≤1−1ηL·log⁡ηR+1ηL·log⁡ηL,

where inf_*n*_(·) is the minimum element of set of *n*. Generally, *E*(*q*∣*k* = *L*) and *E*(*q*∣*k* = *R*) may be different. Since the stopping time is a key point in the sequential analysis, we constrain the two thresholds by unifying *E*(*q*∣*k*) of two classes to be equal, that is, *E*(*q*∣*k* = *L*) = *E*(*q*∣*k* = *R*)≜*q*_*E*_. Then the thresholds are given by

(22) ξL=∑i=1qEEJi ∣ k=L,ξR=∑i=1qEEJi ∣ k=R.

For any given stopping time *q*_*E*_, there is a corresponding threshold pair *ξ*_*L*_ and *ξ*_*R*_. The decision rule with two thresholds *ξ*_*L*_ and *η*_*R*_ is

(23) if  Lq≥ξL,k^=L,if  Lq≤ξR,k^=R,if  ξR<Lq<ξL,compute  Lq+1.

From this decision policy, we can see that other than assigning one of the two classes *L* and *R*, the decision functions may still be undecided and continue testing to the next observation. The “undecided” response keeps the number of errors (false positives or false negatives) low, which is useful for avoiding making excessive mistakes to speed up decisions, for example, a BCI control wheelchair running into an obstacle [16]. In addition, when it is still undecided when reaching to the stopping time *q*_*E*_, we specify that when *q* = *q*_*E*_, the decision rule is

(24) if  Lq>0,k^=L,if  Lq<0,k^=R.

Till now, with the above decision rules, the consequent results, such as accuracy, mutual information (MI) [17], the steepness of MI [18], and average decision time, will only rely on the stopping time *q*_*E*_ and the data to be analyzed. Depending on the actual specific needs, we can set the accuracy, MI, the steepness of MI, and average decision time as the optimization target, respectively, to determine the optimal stopping time *q*_opt_. At the same time, the two thresholds are determined.

## 3. Results

### 3.1. Feature Extraction

To evaluate the performance of our method, we tested it using BCI Competition Datasets II and Dataset IIIb from BCI Competition III. They were obtained from four subjects, denoted as S1–S4. The task performed was based on left and right-hand motor imagination.

**Figure 1:**
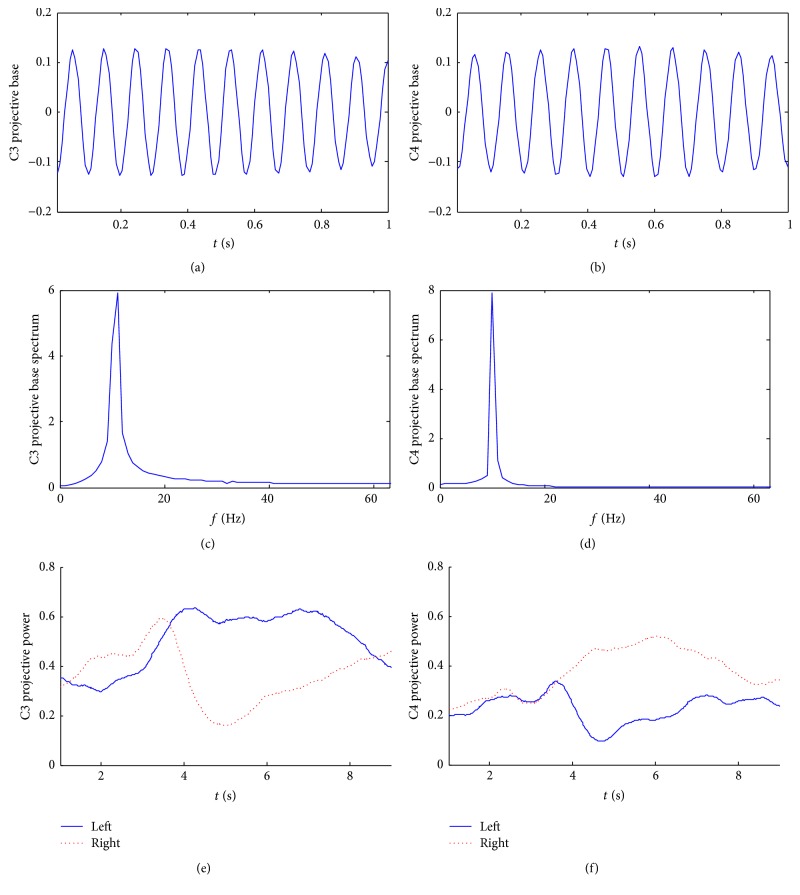
The power projective base of the subject S1. (a) C3 projective base; (b) C4 projective base; (c) C3 projective base spectrum; (d) C4 projective base spectrum; (e) the time-varied projective power of C3; (f) the time-varied projective power of C4.

The dimension of the projective base, that is, the length of the sliding window, is set to be 1 s. The time-domain waveforms of the optimal projective base of the two channels for subject S1 are shown in Figures 1(a) and 1(b). The corresponding frequency spectra are shown in Figures 1(c) and 1(d). The average projective power time courses during the right-hand (dash line) and left-hand (solid line) imagined movement for the C3 and C4 are displayed in Figures 1(e) and 1(f). From this figure, we can see that the projective bases are similar to modulated sine signals and the spectra have band-pass characteristics which are similar to that of wavelet base. For this subject, the projective power dominates in the *μ* rhythm. During the first 3.5 s (0.5 s after cue presentation), the projective power curves under two conditions are close; after 3.5 s, distinct difference in the projective power can be observed which provides a good classification feature. The projective bases for subjects S2 and S3 are similar to that of subject S1.

**Figure 2:**
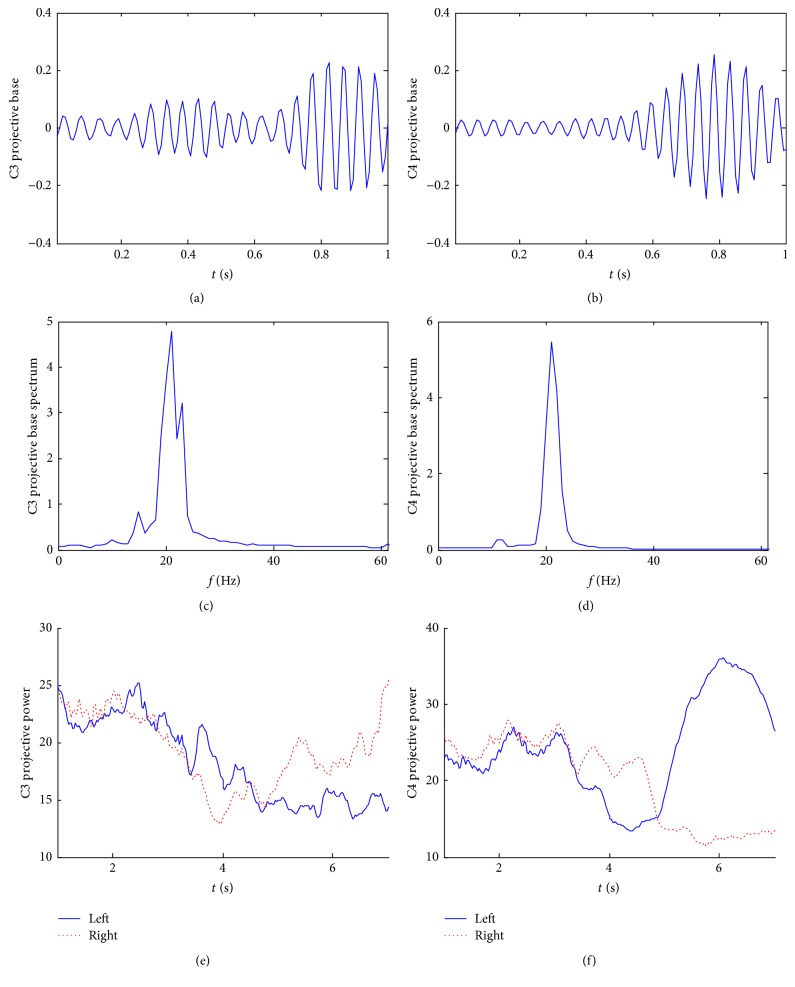
The power projective base of the subject S4. (a) C3 projective base; (b) C4 projective base; (c) C3 projective base spectrum; (d) C4 projective base spectrum; (e) the time-varied projective power of C3; (f) the time-varied projective power of C4.

The power projective base of subject S4 is shown in Figure 2. In contrast with subject S1, the reactivity patterns of the projective bases of this subject are quite different. The waveforms of the projective bases are oscillating faster. Obviously, the frequency of projective power is higher than that of subject S1. As seen in Figures 2(c) and 2(d), the projective bases display a peak in *β* rhythms. Moreover, the results of the average projective power time courses demonstrate that the patterns of ERD/ERS subject S4 are quite different. That means this projective power method is subject-adaptive as well as avoiding the parameter setup in advance.

### 3.2. Classification Results

Two kinds of experiments were performed to evaluate the performance of the proposed machine learning method. One is to evaluate the projective power feature extraction method and the other is to evaluate the SPRT classification performance. In the first one, for the purpose of benchmarking, we compared the classification accuracy (ACC), the mutual information (MI) with two benchmark feature extraction algorithms, and DFT and WT based on the sequential Bayesian classifier [17, 18]. These methods were also applied to the data of subjects S1–S4. The classification accuracy (ACC) and mutual information (MI) of the three methods in consideration are listed in Table 1, where Avg. denotes the averaged indexes over all four subjects. The results of WT are derived from Lemm's method which won the BCI competitions in 2003 and 2005 for motor imagery datasets. From Table 1, we can see that the proposed power projective method outperforms two benchmark methods for every subject. Compared with wavelet method, the average ACC of our method increased from 84.5% to 87.6% and MI increased to 0.468 bits. Furthermore, a paired *t*-test analysis was used to compare the classification accuracies of the three methods. The paired *t*-test result confirms that with projective base method the ACC and MI across subjects are significantly higher than that with WT (*t* = 15.1, *p* < 0.01) and with DFT (*t* = 8.432, *p* < 0.01).

Then we compared the ACC and MI between several state-of-the-art nonsequential classifiers used in the BCI community and their sequential ones with the projective base feature. Those methods were LDA, SVM, and Bayesian, and the corresponding sequential methods were denoted as SLDA, SSVM, and SB. The tenfold cross-validation was carried out for classification tests of EEG data in this study; that is, the datasets for each subject were divided into ten subsets, and the following procedure was repeated ten times. Each time, one of the ten subsets was used as the test set and the other nine were used as the training set. The average recognition rate was evaluated across all ten folds. These methods were applied to the three BCI competition datasets described in Section 2.1 from thirteen subjects S1–S13.

Table 2 shows the classification accuracy of all the methods on the competition test data, where “Avg.” denotes the averaged results over all the subjects. By applying the obtained effective features with the power projective method on the nonsequential methods, a classification accuracy of 76.0%, 71.6%, and 75% was achieved by LDA, SVM, and Bayesian, respectively. The accuracies achieved by all of the corresponding sequential classifiers were greater than the nonsequential one. The paired* t*-test result confirms that with sequential classifier the accuracies across subjects are significantly higher than that with nonsequential ones (*t* = 6.15, *p* < 0.01). Overall, the classification accuracy is higher when the sequential scheme is adopted.

Additionally, it is also important to consider the stopping time for speedy BCI applications without sacrificing accuracy. Therefore, we further analyze the classification accuracy, the MI, and stopping time of the SPRT and compared the time-accuracy trade-off between SPRT and SB. The classification results of the SPRT are provided in Table 3. By applying the obtained effective features with the power projective method on the SPRT, an average classification accuracy of 84.1% was achieved. The proposed SPRT classifier with the projective base features outperforms the SB classifier with the same feature extraction method in terms of classification accuracy across all the subjects except for subject S2. In terms of average accuracy, SPRT outperforms SB classifier. Analyzing the overall results, it can be concluded that SPRT with the projective base feature extraction method outperforms the state-of-the-art methods on the standard datasets.

**Figure 3:**
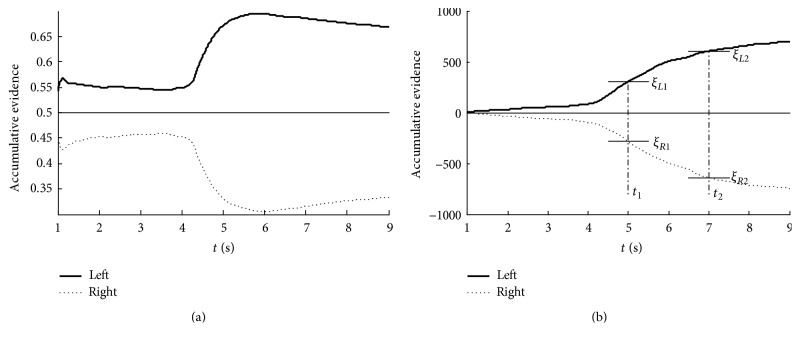
The average accumulative process of classification information for subject S1: (a) SB method; (b) SPRT method.

The resulting time courses of the accumulative classification information, the so-called accumulative evidence, of our SPRT method and SB method are shown in Figure 3.  Figure 3(a) shows that the SB classification method gains information from around 4 s (3 s preparation period and 1 s window length). The cumulative Bayesian posterior probabilities reach the extremum at around 5.5 s, indicating a peak decision confidence at this time. However, the accumulative information falls down at the end of trial. The result shows that the effective control takes place during the middle of a trial. More evidence could not increase the classification performance any further.

Compared with the SB method, the cumulative process of SPRT has monotonicity (shown in Figure 3) which makes it possible to improve the accuracy with more evidence available. During the SPRT classification process, once the accumulative evidence exceeds one of the thresholds, an immediate decision will be given. Moreover, the thresholds can be adjusted to change the decision time. That is to say, with two broader threshold choices, a larger number of observations may be required to improve the accuracy, and vice versa. This can be seen from Figure 3(b), given the expected stopping time *t*_1_, the thresholds are *ξ*_*L*1_, *ξ*_*R*1_. When the expected stopping time is set to be *t*_2_, the thresholds change to be *ξ*_*L*2_, *ξ*_*R*2_. Obviously, it will achieve a higher accuracy with more decision time. This depicts the inherent trade-off between decision time (costs) and accuracy (benefits) of the SPRT method.

**Figure 4:**
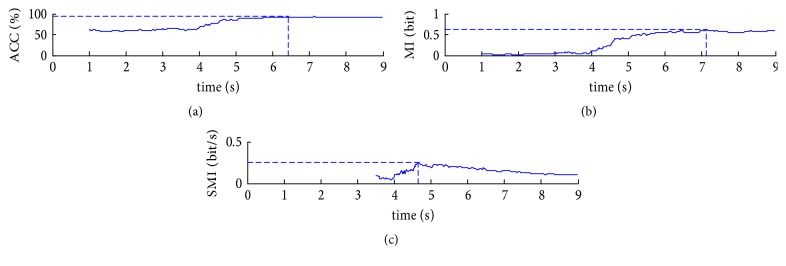
The time course of the classification accuracy, the MI, and the steepness of MI for subject S1: (a) ACC; (b) MI; (c) SMI.

The resulting time courses for the classification accuracy, the MI, and SMI (calculated as MI(*t*)/(*t* − 3 s) for *t* > 3.5 s) for subject S1 are presented in Figure 4. The SMI quantifies the response time. During the first 4 s, the classification performs at a rate no better than chance. Afterwards a steep ascent in the classification accuracy can be observed, meanwhile reflecting a raising MI. The increase of MI indicates an increase in separation ability between left- and right-hand motor imagery. The maximum classification accuracy and the maximum MI are achieved at about 6.4 s and 7.1 s, respectively. The observation of nonmonotonic relationship between accuracy and the time is due to the limited data available which leads to the final decision. With consideration about time, the maximum steepness of MI is obtained at around 4.6 s.

The general sequential framework of the present approach is customizable to suit different task objectives, such as improving accuracy, MI, or steepness of MI. However, the optimization of one particular objective will come at the expense of the others.

## 4. Conclusion

In this paper, we present a SPRT method in conjunction with power projective base method to recognize mental states. The power projective method was first developed to determine features by maximizing the average projection energy difference of the two types of signals. With the accumulative evidence curve, the proposed SPRT sets the two-constrained thresholds based on a desired expected stopping time. The SPRT method adds the benefit of a customizable trade-off between accuracy and decision speed. Specifically, the thresholds in this method were determined without predefined error probabilities. Using standardized datasets, improved performances were demonstrated.

Although this study suggests that SPRT would be useful to balance the speed and accuracy for different BCI applications, we realize that further work requires investigation. Future work will attempt to validate the proposed method with a larger dataset and implement it in our BCI-actuated robotic system. Moreover, we will investigate the multiway SPRT theory and its application to the multiclass BCI systems.

## Figures and Tables

**Table 1:** A comparison of ACC and MI for three methods.

Method	Criterion	S1	S2	S3	S4	Avg.
DFT	Acc (%)	88.6	86.3	76.0	77.9	82.2
	MI (B)	0.488	0.424	0.205	0.238	0.339
WT	Acc (%)	89.3	88.4	79.2	81.1	84.5
	MI (B)	0.509	0.482	0.262	0.301	0.389
PP	Acc (%)	92.1	90.6	83.1	84.4	87.6
	MI (B)	0.601	0.550	0.345	0.375	0.468

**Table 2:** Accuracy (%) of different classifiers on the BCI competition datasets.

Subject	LDA	SLDA	SVM	SSVM	Bayesian	SB
S1	87.8	90.0	87.1	92.1	86.4	92.1
S2	74.6	88.3	74.6	85.7	76.1	90.6
S3	64.4	68.2	64.4	65.4	62.2	83.1
S4	71.8	77.1	66.4	66.4	71.2	84.4
S5	64.0	64.0	60.9	63.1	61.4	66.6
S6	61.2	61.2	59.1	57.1	60.4	64.1
S7	61.1	55.0	61.1	57.6	59.8	65.0
S8	95.7	98.0	87.3	96.0	97.1	98.1
S9	78.2	83.6	73.7	84.2	77.9	85.9
S10	77.5	86.4	66.1	73.5	72.1	80.5
S11	78.8	80.1	76.7	83.1	78.2	79.7
S12	90.4	92.4	80.5	88.4	90.8	92.8
S13	82.8	89.8	72.6	87.7	80.8	86.5

*Avg.*	*76.0*	*79.5*	*71.6*	*76.9*	*75.0*	*82.3*

**Table 3:** The classification results of the SPRT method.

Subject	ACC (%)	MI (bit)	*T* (s)
S1	92.8	0.627	1.23
S2	88.7	0.491	1.95
S3	84.6	0.380	2.80
S4	85.0	0.390	2.35
S5	73.6	0.167	2.33
S6	68.2	0.098	1.23
S7	70.9	0.130	0.87
S8	97.9	0.853	1.33
S9	86.2	0.421	2.48
S10	83.8	0.361	2.54
S11	84.9	0.388	1.76
S12	93.6	0.657	1.69
S13	83.6	0.356	2.23

*Avg.*	*84.1*	*0.409*	*1.91*
